# Evaluation of knowledge, awareness and attitudes towards breast cancer risk factors and early detection among females in Bangladesh: A hospital based cross-sectional study

**DOI:** 10.1371/journal.pone.0257271

**Published:** 2021-09-13

**Authors:** Nur E. Alam, Md. Shariful Islam, Hedayet Ullah, Md. Tarek Molla, Siratul Kubra Shifat, Sumaiya Akter, Salma Aktar, Mst. Mahmuda Khatun, Md. Rayhan Ali, Tapon Chandra Sen, Kamal Chowdhury, Rehana Pervin, A. K. M. Mohiuddin

**Affiliations:** 1 Department of Biotechnology and Genetic Engineering, Mawlana Bhashani Science and Technology University, Tangail, Bangladesh; 2 Department of Biology, University of Kentucky, Lexington, Kentucky, United States of America; 3 Department of Biochemistry and Molecular Biology, Mawlana Bhashani Science and Technology University, Tangail, Bangladesh; 4 Department of Biology, Claflin University, Orangeburg, South Carolina, United States of America; 5 Department of Gynaecology and Obstetrics, Tangail Medical College, Tangail, Bangladesh; Government College University Faisalabad, PAKISTAN

## Abstract

**Background:**

Breast cancer (BCa) is a leading cause of mortality among women in Bangladesh. Many young women in Bangladesh have poor knowledge about breast cancer screening, including risk factors, warning signs/symptoms, diagnosis and early detection. We investigated awareness about breast cancer risk factors as a screening tool among women at the Sheikh Hasina Medical College (SHMC) of Tangail district in Bangladesh.

**Methods:**

A cross sectional survey was conducted to collect data via a structured questionnaire from SHMC during the period of February to December 2019. A total of 1,007 participants (aged 33.47 (±12.37 years)) was considered for data analysis.

**Results:**

Of the 1,007 women, about 50% were knowledgeable about the risk factors. Pain in the breast was identified as the most commonly warning sign/symptom of breast cancer. Only 32.2% of respondents knew at least one breast cancer screening method. The mean knowledge was scored 3.43 ± 2.25 out of a total possible score of 8. Awareness of BCa was associated with residence, family history of breast cancer, marital, literacy and socio-economic status (*p* <0.05). Only 14.7% of women who knew about BSE said they were conducting regular breast self-examination. Unmarried women (aOR: 2.971; 95% CI: 1.108–7.968) were more likely to have performed BSE compared to married women (*p* <0.05).

**Conclusion:**

Although most participants were aware of breast cancer; knowledge about risk factors, warning signs/symptoms, early diagnosis and detection was relatively poor. Knowledge about performing BSE was particularly low. This highlights the importance of increasing awareness about breast cancer risk factors and early detection among young women in Bangladesh.

## Introduction

Breast cancer (BCa) is the most common malignancy that seems to frequently occur with the highest fatality rates in women worldwide [1]. The incidence, morbidity, and mortality rate of breast cancer has been increased in both high and low-resource settings due to the increased life expectancy, urbanization and adoption of western lifestyles [2]. According to global cancer statistics report in 2020, female breast cancer was recognized as the leading cause of global cancer incidence. It has been estimated that about 2.3 million new breast cancer cases were diagnosed, representing about 11.7% of all new cancer cases worldwide [3]. According to the World Health Organization (WHO), an estimated 685,000 females died because of breast cancer in 2020 [4].

Breast cancer becomes epidemic in South Asian countries as the incidence and mortality rate are increasing in a dramatic way. Around 588 million women over 15 years of aged face a rising breast cancer epidemic in these countries. In India, around 100,000 women with breast cancer are diagnosed annually [5] and the mortality rate was 21.5% [6]. In Pakistan 34,066 women were diagnosed in 2018 [7] and the breast cancer mortality rate was 26.76% [8].

Bangladesh is a small and the seventh (nearly 160 million people) most populous country in the world [9]. Recently, the prevalence of breast cancer increasing tremendously but there is no national central cancer registry that can provide the complete nationwide data. Therefore, the actual incidence and mortality of breast cancer is mostly unknown. However, based on cancer registry report 2015–2017 of the National Institute of Cancer Research and Hospital (NICRH), 4930 new breast cancer cases were registered during this period [10]. According to GLOBOCAN, 13,028 new breast cancer cases were diagnosed in 2020, with an age-standardized incidence rate (ASR) of 17 per 100,000 [11]. A report based on the NICRH, the mean age was 41.8 years for the breast cancer patients, maximum (> 56%) cases were among reproductive age women. This presents a higher extent of premenopausal cases and among the patients around 90% are diagnosed at stage III–IV [12]. It might be due to lower preference for the treatment compared to younger family members as only one doctor serves approximately 3,300 people in urban areas and more than 15,000 people in rural areas [13]. Another issue is the socio-cultural factors that contribute to delay in seeking treatment because of breast cancer is a topic that is not freely discussed in public. Besides, scarcity of proper knowledge, low education and ignorance among women are also the major causes behind late detection of breast cancer [14].

The knowledge of risk factors and the early detection methods of breast cancer can successfully reduce the mortality rates and improve the patients’ prognosis. In Bangladesh, Breast self-examination (BSE) could be performed as an effective way for early detection as BSE is simple, inexpensive and more importantly can be carried out by the women themselves in houses [15]. According to the Breast Health Global Initiative (BHGI) reports, if females have adequate knowledge and awareness of breast cancer self-examination (BSE), the disease could be diagnosed at an early stage as well as could be easier to manage the disease [16].

To date, knowledge about breast cancer risk factors, early diagnosis and detection methods has not been assessed among the female population in Tangail district, Bangladesh. Therefore, a hospital-based survey was carried out to explore the scenario of knowledge, awareness and treatment about breast cancer among females in Tangail district of Bangladesh.

## Methods

This cross-sectional study was carried out among 1,100 women in Sheikh Hasina Medical College, Tangail, Bangladesh in the period of February 2019 to December 2019. The respondents were given an explanation of the objectives and benefits of the study. Before the interview, verbal consent was taken from the respondents according to the WHO and Bangladesh Medical Research Council (BMRC) guidelines of ethical consideration. Respondent’s right to refuse and withdraw from study any time was accepted. Confidentiality of the respondents was strictly maintained. As a part of the population-based program, the women’s knowledge, awareness and attitude levels about different aspects of BCa including risk factors, early warning signs, practicing early detection methods, and therapeutic approaches were evaluated in this study.

### Respondents

Participants’ age ranged from 15–75 years with a mean of 33.47 (±12.37) years. Majority (87.8%) of them resided in villages. About 79.3% of participants were married and 73.6% had no formal/primary/secondary education. The majority (88.3%) were Muslims and 55.8% belonged to middle class families (Table 1).

**Table 1:** Socio-demographic characteristics of female respondents (n = 1,007).

Variables	n (%)
**Marital status**	
Married	799 (79.3)
Unmarried	208 (20.7)
**Age**	
15–25	304 (30.2)
26–35	328 (32.6)
36–45	210 (20.9)
46–55	104 (10.3)
56–65	44 (4.4)
66–75	17 (1.7)
Mean ± SD	33.47 ± 12.37
**Living place**	
Rural	884 (87.8)
Urban	123 (12.2)
**Literacy status**	
Undergraduate	194 (19.3)
Graduate	72 (7.1)
Others*	741 (73.6)
**Religion**	
Muslim	889 (88.3)
Hindu	108 (10.7)
Others	10 (1)
**Socio economic status**	
Low	432 (42.9)
Medium	562 (55.8)
High	13 (1.3)

Literacy status: Others* include primary/secondary/no formal education.

### Questionnaire content

Data were collected via a structured questionnaire for this study which was derived from the literature review of the previous peer-reviewed published studies [17–20]. The study questionnaire was first developed in English and translated into Bengali after which translation accuracy was verified by an independent bilingual translator.

The questionnaire was divided into four distinct sections; each one documented with appropriate heading indicating its content. First section contained sociodemographic variables such as marital status, age, literacy, living place, socio-economic status. Second section contained questions about breast cancer’s knowledge. Third and fourth section contained practice status of early detection methods and participants’ risk of breast cancer, respectively.

A knowledge score was calculated by summing the responses for each participant. They were given two points for any two correct responses for each of the four areas. These are: risk factor (such as menarche earlier than normal age, diet and diet related factors, hormones and reproductive factors, and benign breast disease) symptoms (pain in the breast, painless lump in the breast and bloody nipple discharge), early screening methods (such as breast self-examination, clinical breast examination and mammography) and were given two points for any one of the correct responses of treatment associated with breast cancer. The total score ranged between 0 and 8. A total score of 0 to 4 were categorized as insufficient knowledge and a score greater than 4 were considered as sufficient knowledge.

Cronbach’s Alpha was used to assess the reliability coefficient which is a measure of the internal consistency of the questionnaire. The Cronbach’s alpha coefficient was 0.830 for the questionnaire where the value >0.7 is considered acceptable [21].

### Statistical analysis

All the data collected from the survey was entered and analyzed using Statistical packages for social sciences (SPSS) version 20 statistical software. Frequencies, percentages, tables, flow chart were used to describe study variables. Multivariable logistic regression models were generated to assess factors associated with “Performing BSE”. Adjusted odds ratios (aORs) and its 95% confidence intervals (CIs) were estimated. The following variables were adjusted for in the models: marital status, living place, education, socio-economic status and have family history of breast cancer. Collinearity was assessed using the variance inflation factor (VIF) to ensure a strong linear relationship among independent variables included in the model was not present. The goodness of fit of the model was checked using the Hosmer Lemeshow (H-L) test. One-way ANOVA was performed to assess the association between the demographic variables and participants’ overall knowledge of breast cancer. A *p*-value less than 0.05 was considered to be significant.

### Ethical consideration

This study was conducted in accordance with the Declaration of Helsinki. Ethics approval was approved by the Department of Biotechnology and Genetic Engineering, Mawlana Bhashani Science and Technology University, Tangail-1902, Bangladesh (Ref: MBSTU/BGE(Research project(87)/2009(106) Date:29-01-2019). Written permission was also obtained from the Department of Gynecology and Obstetrics, Sheikh Hasina Medical College, Tangail-1900, Bangladesh.

## Results

### Knowledge and awareness about breast cancer and practice of early detection

**Fig 1:**
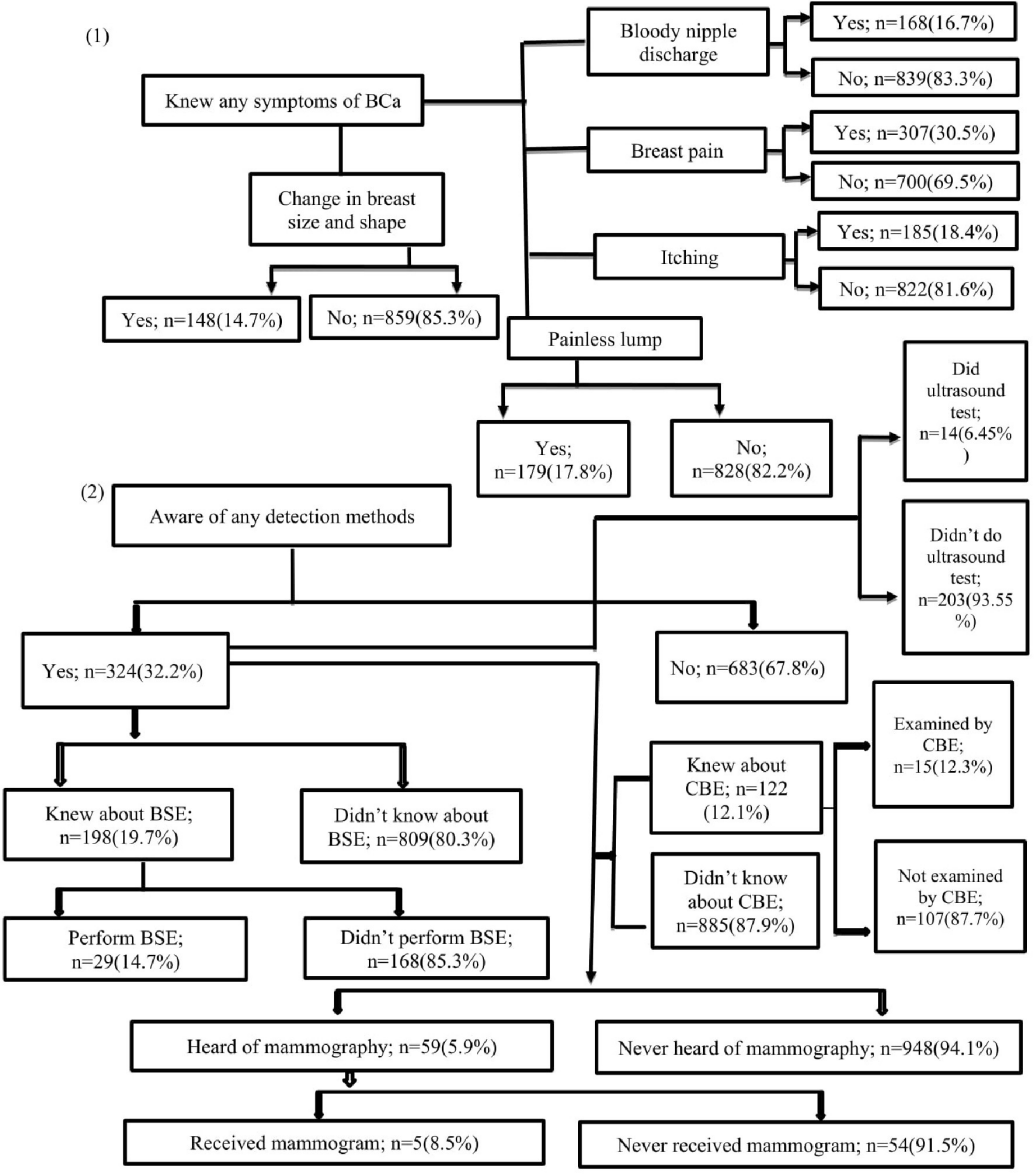
Knowledge and practices of breast cancer and early detection methods.

All of the participants (1007) were familiar with breast cancer. Half of the participants (50%) have knowledge about risk factors of BCa. Menarche earlier than normal age, diet and diet related factors, hormones and reproductive factors, and benign breast disease were mentioned by 23.3%, 19.1%, 16.8% and 15.5% of participants, respectively (Table 2). Like the risk factors, participants were also asked to list at least one symptom of breast cancer had known of or they had heard of. About 30.5% of respondents were aware of that pain in the breast was a major symptom of breast cancer. However, knowledge of other symptoms was generally poor; merely 18.4%, 17.8% and 16.7%, recognized itching, painless lump in the breast and bloody nipple discharge as signs of breast cancer (Fig 1). Majority of the respondents were not aware about other signs of breast cancer like nipple retraction, discoloration of nipple skin, change in breast size, rash around one of the nipples.

**Table 2:** Knowledge about risk factors of breast cancer.

Variable	n (%)
**Have knowledge about risk factors**	
Yes	504 (50)
No	503 (50)
**Diet & diet related factors**	
Yes	192 (19.1)
No	815 (80.9)
**Hormones & reproductive factors**	
Yes	169 (16.8)
No	838 (83.2)
**Benign Breast disease**	
Yes	156 (15.5)
No	851 (84.5)
**Menarche earlier than normal age**	
Yes	235 (23.3)
No	772 (76.7)
**Gender**	
Yes	292 (29)
No	715 (71)
**Obesity**	
Yes	135 (13.4)
No	872 (86.6)

About 32.2% of respondents knew of at least one method of screening for breast cancer including 19.7% knew about breast self-examination (BSE), 12.1% and 5.9% have heard of clinical breast examination (CBE) and mammography. In terms of practice or performing early detection, only 14.7% of women who knew about BSE said they performed it at least once a month. Of those who had heard of CBE and mammography, 87.7% and 91.5% had not received a clinical breast examination or never had received a mammogram (Fig 1).

### Participants risk for breast cancer

**Fig 2:**
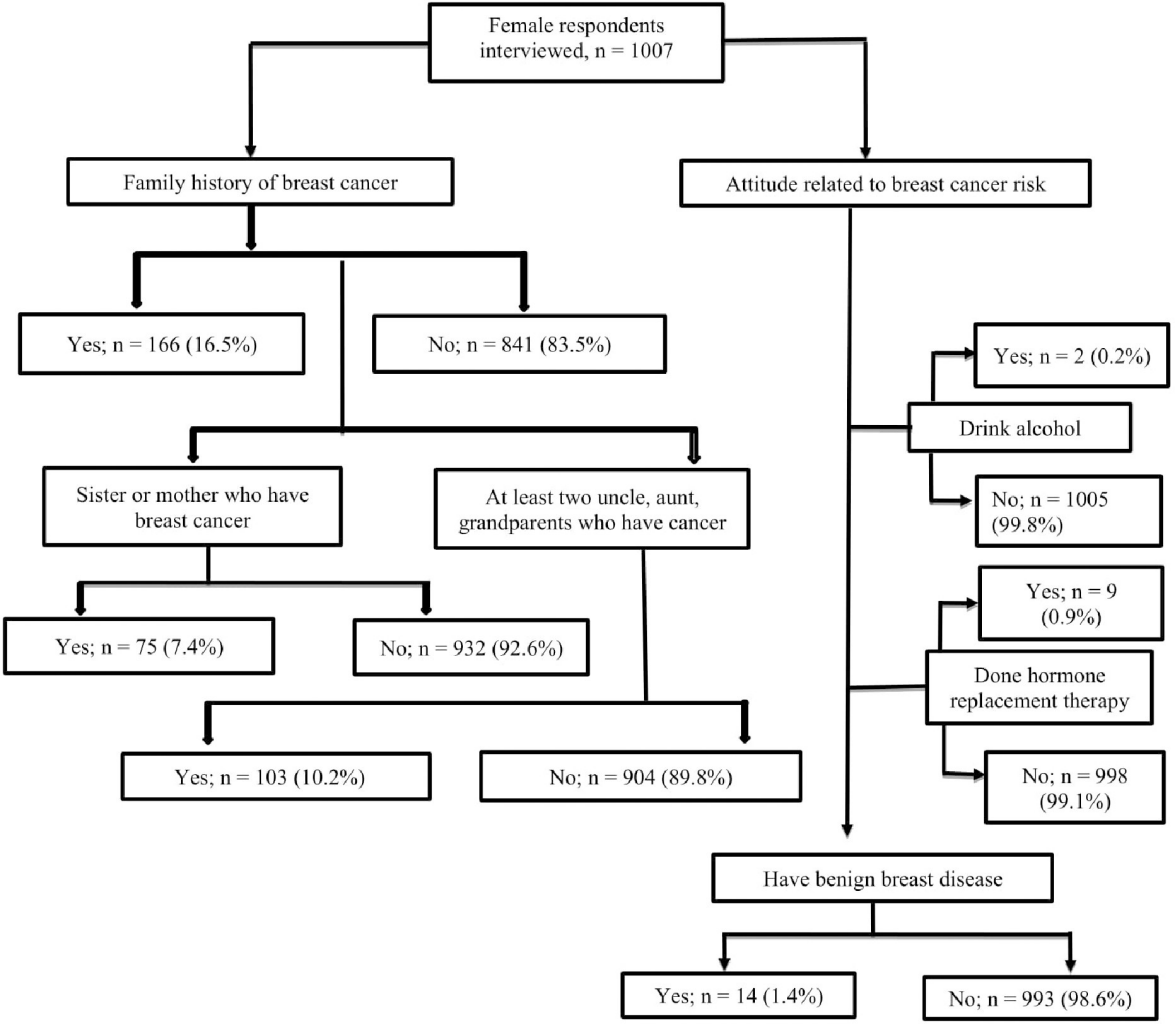
Family history and attitude related to breast cancer of female respondents (1007).

Family history of breast cancer was reported by 166 (16.5%) of the respondents while 7.4% reported that their first-degree relatives (mother, sister) had breast cancer. In addition, 14 (1.4%) of the women have benign breast disease and 9 (0.9%) had performed hormone replacement therapy which may lead to breast cancer (Fig 2).

### Factors associated with knowledge and screening of breast cancer

Table 3 presents factors that may have an impact on acquiring satisfactory knowledge about breast cancer. Among all respondents, 287 (28.5%) had sufficient knowledge of breast cancer. Out of a total possible score of 8, the mean knowledge score was poor (3.43 ± 2.254). Women with a university-level education have better knowledge scores than the women from other education levels. However, women’s knowledge score on breast cancer varied significantly by all the variables except religion (*p*< 0.05).

**Table 3:** Association between demographic variables and total breast cancer knowledge scores (n = 1007).

Variables	Knowledge of breast cancer			*p* value
	Sufficient n = 287 (28.5%)	Insufficient n = 720 (71.5%)	Mean ± SD	
**Marital status**				**<0.001**
Married	193 (24.2%)	606 (75.8%)	3.19 ± 2.167	
Unmarried	94 (45.2%)	114 (54.8%)	4.35 ± 2.346	
**Living place**				**<0.001**
Rural	217 (24.5%)	667 (75.5%)	3.24 ± 2.173	
Urban	70 (56.9%)	53 (43.1%)	4.77 ± 2.374	
**Literacy status**				**<0.001**
Undergraduate	85 (43.8%)	109 (56.2%)	4.12 ± 2.457	
Graduate	31 (43.1%)	41 (56.9%)	4.28 ± 2.563	
Others	171 (23.1%)	570 (76.9%)	3.16 ± 2.105	
**Religion**				0.700
Muslim	259 (29.1%)	630 (70.9%)	3.44 ± 2.293	
Hindu	24 (22.2%)	84 (77.8%)	3.27 ± 1.897	
Others	4 (40%)	6 (60%)	3.70 ± 2.406	
**Socio-economic status**				**<0.001**
Low	97 (22.5%)	335 (77.5%)	3.10 ± 2.044	
Medium	182 (32.4%)	380 (67.6%)	3.64 ± 2.373	
High	8 (61.5%)	5 (38.5%)	5.0 ± 1.958	
**Family history of breast cancer**				**0.001**
Yes	59 (35.5%)	107 (64.5%)	3.96 ± 2.275	
No	228 (27.1%)	613 (72.9%)	3.32 ± 2.236	

Bold shows factors that were significant.

From the multivariable analysis, unmarried women (adjusted odds ratio [aOR]: 2.971; 95% confidence interval [CI]: 1.108–7.968) were more likely to perform BSE compared to married women. We found a significant association between performing BSE and marital status of the women (*p* = 0.031). Women who had primary/secondary/no-formal education (aOR: 0.246; 95% CI: 0.075–0.806) were less likely to have performed BSE. Moreover, middle economic respondents were more likely to perform BSE compared to low and high economic respondents but there was no statistical significance found. The H–L p value for the model was 0.196 (Table 4).

**Table 4:** Factors associated with performing BSE amongst women who had ever heard of BSE (n = 198).

Variables	BSE		Univariate		Multivariate	
	Yes n (%)	No n (%)	OR (95% CI)	*p* value	OR (95% CI)	*p* value
**Marital status**						
Married	12 (9.3)	117 (90.7)	1	**0.004**	1	**0.031**
Unmarried	17 (25)	51 (75)	3.250 (1.447–7.297)		2.971 (1.108–7.968)	
**Living place**						
Rural	21 (14.1)	128 (85.9)	1	0.662	1	0.928
Urban	8 (16.7)	40 (83.3)	1.219 (0.501–2.964)		0.955 (0.354–2.575)	
**Literacy status**						
Undergraduate	13 (22.4)	45 (77.6)	1	**0.013**	1	0.055
Graduate	7 (25.9)	20 (74.1)	0.302 (0.121–0.758)		0.378 (0.129–1.110)	
Others	9 (8)	103 (92)	0.250 (0.083–0.748)		0.246 (0.075–0.806)	
**Socio economic status**						
Low	9 (13.2)	59 (86.8)	1	0.885	1	0.451
Medium	19 (15.7)	102 (84.3)	0.937 (0.103–8.533)		0.731 (0.7–7.572)	
High	1 (12.5)	7 (87.5)	0.767 (0.089–6.595)		1.480 (0.152–14.418)	
**Family history of breast cancer**						
Yes	7 (14)	43 (86)	1	0.868	1	0.747
No	22 (15)	125 (85)	1.081 (0.432–2.709)		0.852 (0.322–2.256)	

## Discussion

This hospital-based study was carried out to determine and assess the knowledge and attitude of breast cancer among females in Bangladesh. Awareness of breast cancer, knowledge, attitudes and regular practice of BSE promote early detection of breast cancer, which improves the chances of survival and better health outcomes. Very few studies have investigated knowledge about breast cancer risk factors and early detection methods among females in Bangladesh, and this is the first study conducted among the general female patients at the Sheikh Hasina Medical College and Hospital of Tangail. Our study provides useful insights to help address this knowledge gap.

In terms of associated risk factors for breast cancer, Half (50%) of the participants had knowledge about risk factors of breast cancer. About 29% of participants claimed that gender (being a woman) was a risk factor for breast cancer followed by menarche earlier than normal age as mentioned by 23.3% of participants. This finding was consistent with a study conducted in Dhaka; Bangladesh that showed 37.5% respondents were aware about breast cancer risk [22]. In contrast to ours, some previous studies reported personal and family history of breast cancer as the most widely known risk factors [2,23–25].

In this study, pain in the breast (30.5%) was reported as the major symptoms of breast cancer by women which indicated that women had inadequate knowledge about breast cancer symptoms. This is consistent with a study conducted in Toronto where 72% of Iranian immigrant women erroneously associated breast pain with early breast cancer [26]. For instance, only a small percentage of women knew that painless breast lump (17.8%) and bloody discharge in nipple (16.7%) as signs of breast cancer that was reported the major symptoms in previous studies performed in different developing countries [15,27,28]. However, in our study other signs of breast cancer like nipple retraction, discoloration of nipple skin, and change in breast size were not recognized by the majority of the women. Similar findings were reported in previous studies from Malaysia showed inadequate knowledge about nipple retraction, discoloration of nipple, breast skin retraction [29,30].

Our study revealed that 16.5% of the respondents had family history of breast cancer while 7.4% reported that their closest relatives (mother, sister) had breast cancer, suggesting a high perceived risk of breast cancer. This study findings were similar to other studies conducted in Bangladesh, Germany, and Saudi Arabia [22,31,32] where 10–25% had at least family members with breast cancer.

In terms of knowledge and attitudes related to breast cancer screening, about one-third of the participants (32.2%) knew about a breast cancer screening method which was lower than a study conducted in Bangladesh that reported 64.2% of participants were aware of the screening methods [18]. Only 19.7% women knew about BSE which is the easiest detection method of breast cancer. The participants who knew about BSE; they (14.7%) hardly practiced it. A recent study from Bangladesh reported that, only 2% respondents mentioned that they were regularly practicing BSE. In addition, it has been reported that, regarding the knowledge about risk factor of breast cancer, 65% respondents have no idea about the risk of breast cancer, where 32% mentioned few risk factors which have relation with breast cancer and 3% respondents did not mention anything [33]. The study findings were consistent with other studies carried out in Sri Lanka [34], Saudi Arabia [35], Nigeria [28] and Ghana [36]. In the current study, the majority of participants who had heard of CBE and mammography reported lower rates of receiving mammograms and clinical breast exams compared with other Asian studies in Turkey, Singapore, and Malaysia [37–39]. From this study finding, the low level of knowledge and practice of breast cancer screening may be one of the main reasons for late presentation of breast cancer among women in Bangladesh.

The results also indicated that the awareness and understanding of breast cancer is associated with educational level, residence, economic status and family history of breast cancer. Unmarried women with high education level and high annual family income tend to be more aware of breast cancer. The practice level for early detection of breast cancer was very low among women which might be due to most of them have no breast cancer symptoms [18].

This study had some limitations that should be considered. The selection of respondents in this study was based on convenience sampling; therefore, our study sample may not be representative of all female population in the Tangail district of Bangladesh. In addition, our findings cannot be generalized to all female patients of Sheikh Hasina Medical College or more widely to others hospitals elsewhere, because potential participants did not have a random chance of being selected. As the survey questionnaire based on multiple choice, it is possible that some respondents might provide socially desirable responses to some questions.

The following initiatives may be useful to prevent and minimize the breast cancer mortality in Bangladesh: Early stage of diagnosis; Using data mining technology to develop risk prediction score software and apps to visualize the risk factor score early; Performing various community campaigns; Preparing nationwide cancer registry report annually and finally Carrying out region-wise epidemiology-based study on a regular basis [40].

## Conclusion

Though this study didn’t cover the whole country, it represents the exact scenario of Bangladeshi women. It is evident that some factors such as education, residential area, and socio-economic status are the main obstacles of being aware about breast cancer. In rural area Bangladeshi women are not getting proper formal education and diagnostic or health care facilities. Lacking of knowledge and awareness about breast cancer along with unavailable diagnostic and treatment facilities are the major reason for breast cancer-related death. This is high time to turn the table by frequently organizing educational programs on breast cancer awareness throughout rural and urban areas. Surely, awareness would lead to early detection and diagnosis, therefore, will improve the odds of survival and cure with simpler and more cost-effective treatment.

## Supporting information

S1 File Breast cancer survey questionnaire. (DOCX)

S2 File Breast cancer SPSS file. (SAV)
