# Supplementary material for: Evaluation of knowledge, awareness and attitudes towards breast cancer risk factors and early detection among females in Bangladesh: A hospital based cross-sectional study
Source: PLoS One. 2021 Sep 13;16(9):e0257271. doi: 10.1371/journal.pone.0257271 (PMC8437277; doi:10.1371/journal.pone.0257271)
Supplement: S1 File — (DOCX) [file pone.0257271.s001.docx]

**Sample ID: Form #**

***Socio‐demographic profile (আর্থ-সামাজিক অবস্থা)***

1. Marital Status (বৈবাহিক অবস্থা**)**

| o Married (বিবাহিত) | o Unmarried (অবিবাহিত) |
| --- | --- |

2. Age (year) **(বয়স)**

| o 15-25 (১৫-২৫) | o 26-35 (২৬-৩৫) | o 36-45 (৩৬ - ৪৫) |
| --- | --- | --- |
| o 46-55 (৪৬-৫৫) | o 56-65 (৫৬-৬৫) | o 66-75 (৬৬ - ৭৫) |

3. Living place (বাসস্থান)

| o Rural (গ্রাম) | o Urban (শহর) |
| --- | --- |

4**.** Study level (শিক্ষাগত যোগ্যতা)

| o Undergraduate (স্নাতকা্থী) | o Graduate (স্নাতক) | o Others (অন্যান্য) |
| --- | --- | --- |

5**.** Religion (ধর্ম)

| o Muslim (মুসলিম) | o Hindu (হিন্দু) | o Others (অন্যান্য) |
| --- | --- | --- |

6. Socio‐economic level **(**আর্থ-সামাজিক অবস্থান**)**

| o Low (নিম্ন) | o Middle (মধ্যম) | o High (উচ্চ) |
| --- | --- | --- |

***Knowledge about Breast Cancer (স্তন ক্যান্সার সম্পর্কে জ্ঞান)***

7. Do you have any knowledge about the risk factors of breast cancer? (আপনি কি স্তন ক্যান্সারের ঝুঁকিপূর্ণ কারণগুলি সম্পর্কে জানেন?)

| o Yes (হ্যাঁ) | o No (না) |
| --- | --- |

8. Do you know about any of the following risk factors of breast cancer? (স্তন ক্যান্সারের নিম্নলিখিত ঝুঁকির সম্পর্কে জানেন?)

| Diet & diet related factors (খাবার এবং খাবার সম্পর্কিত) | o Yes (হ্যাঁ) | o No (না) |
| --- | --- | --- |
| Hormones & reproductive factors (হরমোন এবং প্রজনন সম্পর্কিত) | o Yes (হ্যাঁ) | o No (না) |
| Ionizing radiation (বিকিরণ সম্পর্কিত) | o Yes (হ্যাঁ) | o No (না) |
| Benign Breast disease (স্তনে টিউমার জাতীয় রোগ) | o Yes (হ্যাঁ) | o No (না) |
| Menarche earlier than normal age (অল্প বয়সে ঋতুস্রাব) | o Yes (হ্যাঁ) | o No (না) |
| Gender (লিঙ্গ) | o Yes (হ্যাঁ) | o No (না) |
| Obesity (স্থূলত্ব) | o Yes (হ্যাঁ) | o No (না) |
| Hormone replacement therapy (হরমোনের সাহায্যে চিকিৎসা) | o Yes (হ্যাঁ) | o No (না) |

9. Do you have heard about the early detection methods of Breast Cancer? *(আপনি কি কখনও স্তন ক্যান্সারের প্রাথমিক সনাক্তকরণ পদ্ধতি সম্পর্কে শুনেছেন?)*

| o Yes (হ্যাঁ) | o No (না) |
| --- | --- |

10. Do you have any knowledge about any of the following detection methods of Breast Cancer? (স্তন ক্যান্সারের নির্ণয় সম্পর্কে নিম্নলিখিত কোনও পদ্ধতি আপনার কি জানা আছে?)

| BSE (Breast Self ‐Examination) (নিজে পরীক্ষা করে দেখা) | o Yes (হ্যাঁ) | o No (না) |
| --- | --- | --- |
| CBE (Clinical Breast Examination) (ক্লিনিক্যাল পরীক্ষা) | o Yes (হ্যাঁ) | o No (না) |
| Mammography (MMG) (ম্যামোগ্রাম) | o Yes (হ্যাঁ) | o No (না) |
| Ultrasound for detection of breast tumor (আল্ট্রাসাউন্ড করে টিউমার পরীক্ষা করা) | o Yes (হ্যাঁ) | o No (না) |
| Breast MRI scan (স্তন এম.আর.আই. করা) | o Yes (হ্যাঁ) | o No (না) |

11. Do you know about any of the following symptoms of Breast Cancer? (নিচের কোনো উপসর্গ সম্পর্কে জানেন কি ?)

| Nipple retraction/inversion (স্তনবৃন্ত প্রত্যাহার / বিপর্যয়) | - Yes (হ্যাঁ) | - No (না) |
| --- | --- | --- |
| Breast pain (স্তনে ব্যাথা) | - Yes (হ্যাঁ) | - No (না) |
| Breast skin change (চামড়া পরিবর্তন) | - Yes (হ্যাঁ) | - No (না) |
| Bloody nipple discharge (স্তনবৃন্ত থেকে রক্ত নিঃসৃত) | - Yes (হ্যাঁ) | - No (না) |
| Painless lump in one of the armpits/breast (স্তনে ব্যথাহীন ছোপ) | - Yes (হ্যাঁ) | - No (না) |
| A rash around one of the nipples (স্তনবৃন্ত চুলকানো) | - Yes (হ্যাঁ) | - No (না) |
| Redness, discoloration of the nipple skin (স্তনবৃন্ত লাল হয়ে যাওয়া) | - Yes (হ্যাঁ) | - No (না) |
| Change in Breast size & shape (স্তনআকৃতি পরিবর্তন) | - Yes (হ্যাঁ) | - No (না) |
| Itching (চুলকানো) | - Yes (হ্যাঁ) | - No (না) |

12. Do you have any knowledge about any one of the following Breast Cancer treatments? *(নিচের কোনো চিকিৎসা পদ্ধতি সম্পর্কে জানা আছে ?)*

| - Chemotherapy (কেমোথেরাপি) | - Surgery (সার্জারি) | - Biological(drug) therapy (ওষুধ) |
| --- | --- | --- |
| - Radiotherapy (রেডিওথেরাপি) | - Synthetic Breast transplant (কৃত্রিম স্তন সংযোজন) | - Hormone therapy (হরমোন থেরাপি) |
| - Do not know (জানি না) |  |  |

***Practice status of early detection method of Breast Cancer (স্তন ক্যান্সারের প্রাথমিক সনাক্তকরণ পদ্ধতি অনুশীলন)***

13. Did you perform BSE (Breast Self‐ Examination) at least once a month? *(আপনি কি মাসে কমপক্ষে একবার নিজে স্তন পরীক্ষা করেছিলেন?)*

| o Yes (হ্যাঁ) | o No (না) |
| --- | --- |

14. Did you perform CBE (Clinical Breast Examination) within last one year? (আপনি কি গত এক বছরের মধ্যে ক্লিনিকাল ব্রেস্ট পরীক্ষা করেছেন?)

| o Yes (হ্যাঁ) | o No (না) |
| --- | --- |

15. Did you perform Mammography in the past? (আপনি অতীতে ম্যামোগ্রাফি করেছেন?)

| o Yes (হ্যাঁ) | o No (না) |
| --- | --- |

16. Did you perform Ultrasound test procedure within last one year? (আপনি কি এক বছরের মধ্যে আল্ট্রাসাউন্ড পরীক্ষাটি করেছিলেন?)

| o Yes (হ্যাঁ) | o No (না) |
| --- | --- |

***Participants risk for Breast Cancer (অংশগ্রহণকারীরা যাদের স্তন ক্যান্সারের ঝুঁকি আছে)***

17. Do you have family history of Breast Cancer? (আপনার পরিবারে কারও স্তন ক্যান্সার আছে?)

| o Yes (হ্যাঁ) | o No (না) |
| --- | --- |

18. Do you have any sister or mother suffering from breast cancer? (আপনার কি কোনও বোন বা মা স্তন ক্যান্সারে ভুগছেন?)

| o Yes (হ্যাঁ) | o No (না) |
| --- | --- |

19. Do you have at least two uncles, aunt, grandparents who have cancer? (আপনার কি কমপক্ষে দুজন চাচা, খালা, দাদা-দাদি যাদের ক্যান্সার রয়েছে?)

| o Yes (হ্যাঁ) | o No (না) |
| --- | --- |

20. Do you drink Alcohol? (আপনি কি অ্যালকোহল পান করেন?)

| o Yes (হ্যাঁ) | o No (না) |
| --- | --- |

21. Have you done any Hormone replacement therapy? (আপনি কি কোন হরমোন প্রতিস্থাপন থেরাপি নিয়েছেন ?)

| o Yes (হ্যাঁ) | o No (না) |
| --- | --- |

22. Do you have any benign breast disease? (আপনার কি স্তনে টিউমারজনিত রোগ আছে?)

| o Yes (হ্যাঁ) | o No (না) |
| --- | --- |
